# Ectopic ACTH Production in Medullary Thyroid Carcinoma—A Study of Two Cases

**DOI:** 10.1155/crie/8889322

**Published:** 2026-07-07

**Authors:** Robert Bränström, Fredric Hedberg, Monique Huisman, Maria Petersson, Camilla Jerning, Ivan Shabo, David J. M. Goldstein, Martin Larsson, C. Christofer Juhlin

**Affiliations:** ^1^ Department of Breast, Endocrine Tumors and Sarcoma, Karolinska University Hospital, Stockholm, Sweden, karolinska.se; ^2^ Department of Molecular Medicine and Surgery, Karolinska Institutet, Stockholm, Sweden, ki.se; ^3^ Department of Endocrinology, Karolinska University Hospital, Stockholm, Sweden, karolinska.se; ^4^ Department of Surgery, Medisch Spectrum Twente, Enschede, Netherlands, mst.nl; ^5^ Department of Oncology-Pathology, Karolinska Institutet, Stockholm, Sweden, ki.se; ^6^ Department of Pathology and Cancer Diagnostics, Karolinska University Hospital, Stockholm, Sweden, karolinska.se

**Keywords:** Cushing’s syndrome, ectopic aCTH production, medullary thyroid cancer, targeted therapy, tyrosine kinase inhibitors

## Abstract

Medullary thyroid carcinoma (MTC) is a rare neuroendocrine tumor originating from thyroid parafollicular C‐cells, accounting for 1–2% of all thyroid cancers. An exceedingly rare manifestation of MTC is ectopic adrenocorticotropic hormone (ACTH) production, causing Cushing’s syndrome and complicating management. This report presents two cases of MTC with ectopic ACTH production, highlighting diagnostic challenges, therapeutic strategies, and clinical outcomes. A comprehensive literature review on this rare paraneoplastic syndrome is included and supplements the case findings. Case 1 involves a 49‐year‐old man presenting with abdominal pain, weight loss, and pulmonary nodules, diagnosed with MTC and ectopic ACTH‐related Cushing’s syndrome. Surgical resection and targeted therapy with selpercatinib improved cortisol levels but were complicated by adverse drug reactions. Case 2 details a 65‐year‐old woman with severe hypercortisolism and locally advanced MTC. Selpercatinib successfully reduced hormone levels and achieved partial tumor regression. Both cases underscore the critical role of tyrosine kinase inhibitors (TKIs) in controlling tumor progression, and paraneoplastic hormone production is exemplified in both cases, as treatment initiation was followed by a biochemical response with declining levels of ACTH, cortisol, and calcitonin. Ectopic ACTH production in MTC is a rare but clinically significant entity associated with aggressive disease. Early recognition, comprehensive biochemical and imaging evaluations, and a multidisciplinary approach are pivotal for optimal management. The advent of targeted therapies, such as selpercatinib, has transformed the therapeutic landscape, offering improved control of both tumor burden and hormone excess. This report highlights the importance of integrating genomic insights and precision medicine in addressing these complex cases.

## 1. Introduction

Medullary thyroid carcinoma (MTC) is a rare but distinct form of thyroid malignancy originating from the parafollicular C‐cells of the thyroid gland. Unlike the more common follicular‐cell‐derived thyroid cancers, MTC accounts for ~1%–2% of all thyroid cancer cases [1, 2] and is classified into sporadic and hereditary forms, such as multiple endocrine neoplasia type 2 (MEN2) syndrome [1].

MTC poses a significant clinical challenge due to its variable biological behavior and the potential for early metastasis. The tumor’s propensity to secrete calcitonin and carcinoembryonic antigen (CEA) constitutes a diagnostic marker and a tool for monitoring disease progression and response to treatment [2]. Despite advances in the early detection and management of thyroid cancers, MTC often presents at a more advanced stage, contributing to its poorer prognosis [2].

MTC is associated with genetic mutations in pathways involved in its pathogenesis. The *RET* proto‐oncogene mutation is central to developing sporadic and hereditary MTC, with specific mutations correlating with the disease phenotype and aggressiveness. This has paved the way for targeted therapies, such as tyrosine kinase inhibitors (TKIs) vandetanib and cabozantinib, which inhibit multiple pathways, including *RET* signaling, offering promising outcomes for patients with advanced or metastatic MTC [3]. The *RET* wildtype MTCs are usually driven by oncogenic mutations in *RAS* family genes in a mutually exclusive fashion [4].

The integration of genomic profiling into clinical practice refines therapeutic strategies, allowing for a personalized approach to treatment. This precision medicine paradigm is anticipated to improve outcomes by tailoring interventions to each patient’s tumor’s genetic and molecular landscape [5].

The MTC is a complex disease requiring a multidisciplinary approach to diagnosis and management. Integrating molecular genetics into clinical practice has revolutionized the management and treatment of this disease.

Beyond calcitonin and CEA secretion, MTC can occasionally exhibit neuroendocrine features, including rare hormone‐producing capabilities. Among these rare manifestations, ectopic ACTH production leading to Cushing’s syndrome is exceptionally uncommon, occurring in fewer than 1% of MTC cases. The clinical presentation and management of this paraneoplastic phenomenon are not well characterized, with only a limited number of cases reported in the literature [6–9]. Hence, we present two cases of MTC associated with the unexpected and rare condition of suspected ectopic ACTH‐related Cushing’s syndrome, detailing the clinical findings, management, and outcomes.

## 2. Case 1

**Figure 1:**
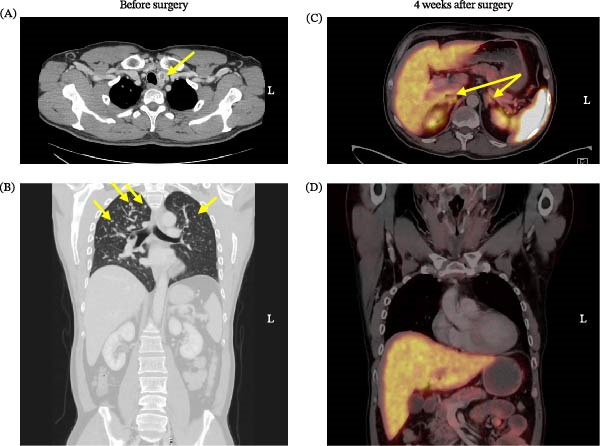
Selected radiological examinations of Case 1. CT images (A,B) prior to surgery reveal a tumor mass in the left thyroid lobe and multiple lung metastases. Yellow arrows indicate the left‐sided thyroid tumors in panel A and selected lung metastases in panel B. Approximately 4 weeks after thyroidectomy (C,D), a ^68^Ga‐DOTATOC‐PET/CT scan (C,D) was performed, showing no radiotracer‐positive tumors in the neck, lungs, or liver, but enlarged adrenals bilateral indicated with yellow arrow (C). Transverse (A and C) and coronal (B and D) sections are presented, and “L” indicated left side.

A 49‐year‐old previously healthy man presented to his general practitioner in April 2024 with abdominal pain, diarrhea, and 6‐kg weight loss. Initial clinical examination and routine blood tests were normal. Computer tomography (CT) of the chest and abdomen revealed miliary nodules in the lungs, raising suspicion of either metastases or tuberculosis (Figure 1A,B).

The patient was admitted to the department of infectious diseases, and further investigations, including a bronchoscopy, revealed no abnormalities. Cytology indicated inflammation but showed no malignant cells and no signs of tuberculosis. Given that the CT scan also showed colon thickening and a contrast‐enhancing nodule in or near the thyroid gland (Figure 1A), further investigations were conducted, including a colonoscopy, which was normal except for small benign polyps. Ultrasound of the thyroid revealed several pathological lymph nodes in region 4. On the left side of the thyroid, two low‐echogenic, non‐calcified nodules were identified, measuring 10 × 9 × 6 mm and 30 × 13 × 28 mm. Both were poorly defined and classified as TI‐RADS 5. Ultrasound‐guided fine needle aspiration (FNA) of the thyroid revealed MTC. The suspicion of MTC was not incidental but was based on recognizable histomorphological features identified on routine hematoxylin‐eosin examination. The tumor cells exhibited finely granular cytoplasm and characteristic salt‐and‐pepper chromatin, a constellation well established as suggestive of neuroendocrine differentiation and MTC specifically. On this basis, targeted immunohistochemistry, including calcitonin and TTF‐1 was applied for confirmation, appropriately driven by morphological pattern recognition rather than fortuitous selection.

The blood samples showed elevated serum calcitonin and CEA levels (2850 pmol/L; reference (ref.) <3.5 pmol/L and CEA 49 µg/L; ref. <4.7 µg/L, respectively). Metanephrine, normetanephrine, and 3‐methoxytyramine were all within normal limits: 0.2 nmol/L (ref. <0.3), 0.4 nmol/L (ref. <0.8), and <0.1 nmol/L (ref. <0.2), respectively. See Table 1 for a summary of laboratory findings at the time of diagnosis for both Case 1 and Case 2.

**Table 1:** Summary of key‐laboratory findings at the time of diagnosis in Case 1 and Case 2.

Laboratory parameter	Reference range	Case 1	Case 2
Calcitonin (pmol/L)	<3.5	2850	8460
CEA (µg/L)	<4.7	49	700
ACTH (pmol/L)	1.6–14	173	38
Cortisol (nmol/L)	70–330	1452	1255
Urinary cortisol (nmol/24 h)	40–170	3350	Not measured
Dexamethasone suppression (cortisol, nmol/L)	<50	2043	980
Metanephrine (nmol/L)	<0.3	0.2	<0.2
Normetanephrine (nmol/L)	<0.8	0.4	0.4
3‐Methoxytyramine (nmol/L)	<0.2	<0.1	<0.1
Glucose	—	Elevated	Slightly elevated

*Note:* Reference ranges are provided where applicable. All values reflect measurements prior to initiation of treatment/surgery.

Subsequent examination with an ultrasonography of the neck showed left lateral lymphadenopathy with FNA confirming lymph node metastases. The patient underwent total thyroidectomy with a central and left‐lateral lymph node dissection in July the same year. Perioperatively, the patient had remarkable hypertension, polyuria (2.7 L), and hypokalemia (2.1 mmol/L). Targeted panel analysis of blood‐derived DNA, focusing on endocrine disorders, did not identify any pathological findings associated with MEN. Biochemical examination revealed no sign of pheochromocytoma with normal preoperative methoxylated catecholamine levels. Although clinical examination revealed no overt signs of Cushing’s syndrome, both serum ACTH and cortisol levels were markedly elevated—ACTH at 173 pmol/L (reference: 1.6–14 pmol/L) and cortisol at 1452 nmol/L (reference: 70–330 nmol/L). These findings led to a clear suspicion of ectopic Cushing’s syndrome secondary to MTC. The diurnal ACTH/cortisol curve and 24‐h urinary cortisol excretion were abnormal, whereas salivary cortisol was not measured. Following an overnight high‐dose (8 mg) dexamethasone suppression test, serum cortisol remained high at 2043 nmol/L. A ^68^Ga‐DOTATOC‐PET/CT scan showed no pathological radionucleotide uptake in the neck or lungs (Figure 1C,D), but it revealed enlarged adrenal glands compared to the index CT scan (Figure 1C). Neither pituitary MRI nor corticotropin‐releasing hormone (CRH) stimulation testing was performed. In addition to hypertension and hypokalemia, the patient developed diabetes mellitus.

**Figure 2:**
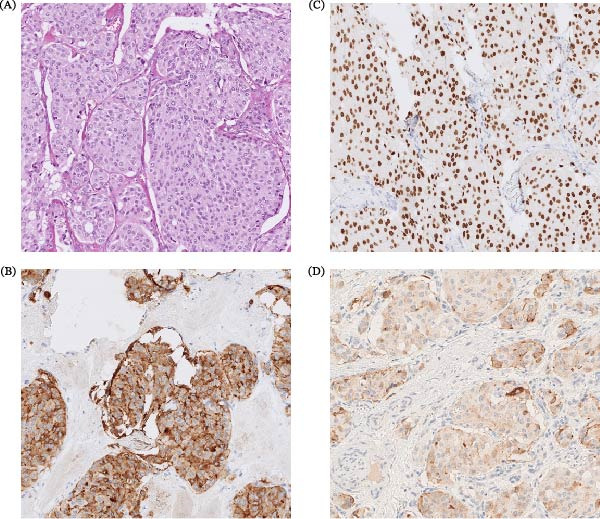
Histological and immunohistochemical characteristics of the thyroid tumor in lymph node metastasis of Case 1. Hematoxylin and eosin (H and E) stainings reveal tumor cells arranged in large nests separated by delicate fibrous septa (A). The tumor cells exhibit amphophilic granular cytoplasm and monomorphic nuclei. Immunohistochemical analysis confirmed diffuse nuclear expression of thyroid transcription factor 1 (TTF‐1) (B), widespread cytoplasmic immunoreactivity for calcitonin (C), and weak background cytoplasmic ACTH immunostaining with focal areas of higher‐intensity positivity (D). These findings support the diagnosis of medullary thyroid carcinoma with ectopic ACTH expression. Weak and focal ACTH immunoreactivity, as observed here, is consistent with previous reports on ectopic ACTH‐producing neuroendocrine tumors, including MTC [9–11].

Upon confirmation of Cushing’s syndrome, treatment with metyrapone was initiated at a starting dose of 500 mg three times daily, and the dose was gradually increased over the following weeks to 1250 mg three times daily. Hypertension was managed with metoprolol and spironolactone, diabetes was treated with sitagliptin, and hypokalemia was corrected with spironolactone and potassium chloride. The postoperative histopathologic examination confirmed the presence of two primary tumors in the left thyroid lobe: an 11 mm high‐grade MTC with Ki‐67 index of 26% and an adjacent 1.6 mm low‐grade MTC. Extensive lymph node metastases were identified in central and lateral compartments, of which the largest metastasis showed focal ACTH immunoreactivity (Figure 2). It is well‐documented that immunohistochemical expression of ACTH in ectopic ACTH‐producing MTC is frequently weak and focal in nature [10–12]. Molecular analysis revealed a somatic mutation in the *RET* gene, exon 16, c.2753T >C, p.Met918Thr. However, genetic testing using an endocrine panel including MEN1 and MEN2 did not detect any germline mutation, supporting a sporadic form of the disease.

All ACTH stainings (both Case 1 and Case 2) were performed using a standardized, accredited clinical protocol with a monoclonal antibody and automated staining on a Ventana platform. External quality controls were included for every case, sectioned and mounted on the same slide as the target tissue. These consisted of lymph node and colon tissue as negative controls and ACTH‐positive pituitary neuroendocrine tumor tissue as a positive control. Only stainings demonstrating appropriate control performance were accepted for interpretation. Full procedural parameters and antibody information are maintained within the clinical protocol and can be provided upon reasonable request.

In August, selpercatinib 160 mg twice daily was introduced, resulting in an immediate decrease in cortisol levels to 54 nmol/L and improved potassium levels, blood pressure, and plasma glucose. Hydrocortisone (10 mg) was added three times daily and the metyrapone dose was reduced from 1250 to 750 mg three times a day. Unfortunately, the patient developed severe skin side effects, necessitating the discontinuation of selpercatinib after 1 week. Cortisol levels rapidly rebounded to 1423 nmol/L and the patient regained Cushing‐related symptoms. The metyrapone dose was increased to 1000 mg thrice daily, and hydrocortisone was reduced to 20 mg daily. The approximately 4‐month interval between initial presentation in April and the initiation of selpercatinib in August reflected several concurrent factors: the sequential nature of the diagnostic workup, reduced institutional capacity during the summer period, the time required to obtain somatic RET mutation results, and the need to stabilize hypercortisolism prior to initiating systemic oncological therapy.

**Figure 3:**
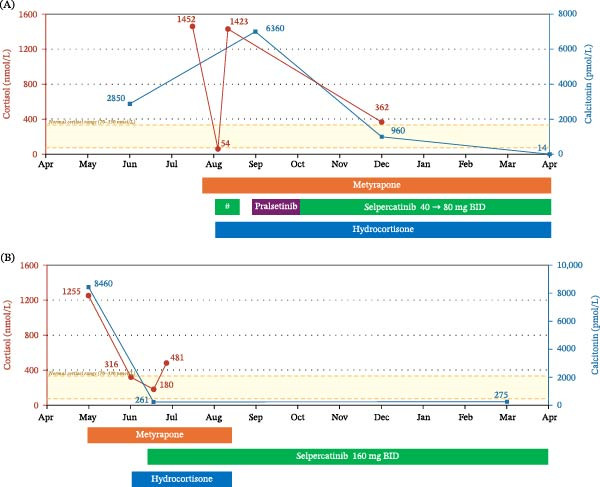
Timeline showing key values for Case 1 (A) and Case 2 (B). Yellow band = normal serum cortisol reference range (70–330 nmol/L). BID, bis in die (twice daily); Numeric values at data points in bold. # Selpercatinib 160 mg was discontinued after 1 week due to severe skin adverse reaction.

The patient started pralsetinib 400 mg once daily; however, cortisol levels and Cushing‐related symptoms did not improve. After discussion at the Swedish National MDT conference, the treatment by pralsetinib was replaced by selpercatinib at a reduced dose (40 mg twice daily). After 2 weeks without adverse effects, the dose was increased to 80 mg twice daily in October. This adjustment decreased cortisol levels to 362 nmol/L, allowing for the tapering of metyrapone, with serum calcitonin levels at 969 pmol/L, respectively, in December compared to 6960 pmol/L 3 months before. And CEA 73 μg/L compared to 95 μg/L. A CT scan of the neck, thorax, and abdomen performed in December showed partial regression of lung metastases compared to the PET/CT scan in July. Magnetic resonance imaging (MRI) of the pituitary revealed no evidence of an adenoma. At 6‐month follow‐up, the response persisted on the CT scan, with CEA and calcitonin levels reduced to 62 µg/L and 14 pmol/L, respectively. A clinical timeline summarizing the key diagnostic and therapeutic milestones alongside longitudinal changes in cortisol and calcitonin levels is provided in Figure 3.

## 3. Case 2

A 65‐year‐old woman with a history of untreated psoriasis presented to the emergency room in May 2024. She reported progressive weakness, fatigue, proximal muscle weakness, diarrhea, and a 20‐kg weight loss over the past 6 months. Physical examination revealed a left‐sided Virchow’s node and a dorsocervical hump. Additional findings included hypertension, perioral hirsutism, pitting edema, and psoriatic plaques on the lower limbs.

Initial laboratory tests revealed hypochloremic metabolic alkalosis and electrolyte disturbances, including severe hypokalemia (1.5 mmol/L; ref. 3.5–4.6 mmol/L), hypocalcemia, hypophosphatemia, and mild hypermagnesemia. Slightly elevated glucose levels were also noted. The working diagnosis was a malignancy with ectopic Cushing’s syndrome. She was admitted to the intermediate care unit for treatment of the electrolyte disturbances and hypertension with intravenous potassium infusions and other medication, including spironolactone.

Morning serum cortisol was elevated to 1255 nmol/L (ref. 135–540 nmol/L). An increased plasma ACTH level of 38 pmol/L (ref. 1.6–14 pmol/L) indicated ACTH‐dependent cortisol production. A 1 mg overnight dexamethasone suppression test failed to suppress cortisol levels (980 nmol/L), supporting the diagnosis of Cushing’s syndrome. Further imaging, including CT of the abdomen and MRI of the pituitary, did not identify a primary source. Analysis of methoxycatecholamine was normal; see Table 1 for a summary of laboratory findings. Given the severity of symptoms due to hypercortisolism, treatment with metyrapone was initiated at an initial dose of 250 mg twice daily and subsequently escalated to 500 mg three times daily within 5 days. The urgency of the patient’s condition did not allow for postponement of therapy to complete the full diagnostic evaluation for ACTH‐dependent Cushing’s syndrome, including 24‐h urinary free cortisol, inferior petrosal sinus sampling, CRH stimulation, or high‐dose dexamethasone suppression testing. As a result, definitive differentiation between pituitary and ectopic ACTH production was not possible at this stage.

With metyrapone treatment, morning serum cortisol gradually decreased to 316 nmol/L, and hydrocortisone 10 mg twice daily was added as a block‐and‐replace regimen.

**Figure 4:**
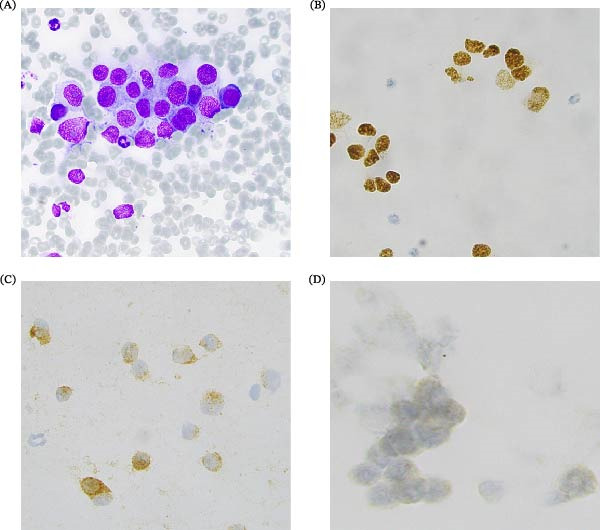
Fine‐needle aspiration cytology from the thyroid (Case 2). May‐Grünwald‐Giemsa (MGG) staining shows tumor cells with a high nuclear‐to‐cytoplasmic ratio and eccentric nuclei displaying characteristic salt‐and‐pepper chromatin (A). Immunocytochemistry reveals diffuse nuclear positivity for thyroid transcription factor 1 (TTF‐1) (B), strong cytoplasmic expression of synaptophysin (C), and focal, weak background cytoplasmic ACTH immunostaining with focal areas of stronger positivity (D). Images are shown at 400× magnification, except for (D), which is at 600× magnification.

**Figure 5:**
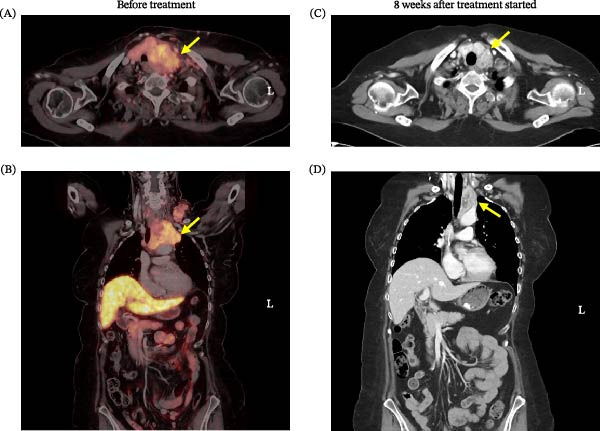
Radiological examinations of Case 2. ^68^Ga‐DOTATOC‐PET/CT scans (A,B) prior to the start of treatment with TKI reveal large tumors on the left side of the thyroid, along with extensive lymph node metastases in ipsilateral regions 2–4. A CT scan performed 8 weeks after the start of treatment is shown, with an overall reduction in size of the tumors indicating a partial radiological response. Transverse (A,C) and coronal (B,D) sections are presented, with “L” indicating the left side. Yellow arrows indicate the thyroid tumor.

A CT scan of the neck, thorax, and abdomen revealed a large mass in the left thyroid lobe extending into the upper mediastinum, with tracheal deviation, infiltration of the esophagus, and encasement of the left common carotid artery and internal jugular vein. Metastatic cervical lymph nodes were observed in left cervical regions 2–5. There were no signs of distant metastases. The patient’s symptoms of flushing, diarrhea, and hypertension raised suspicion of MTC with ectopic ACTH production. Additional laboratory tests confirmed elevated serum calcitonin and CEA (8460 pmol/L and 700 µg/L, respectively). FNA cytology of the thyroid confirmed MTC (Figure 4). Small cell lung cancer (SCLC) was considered early in the differential diagnosis given the mediastinal extension and severity of hypercortisolism, as SCLC is among the most common causes of ectopic ACTH syndrome. However, several features argued against a pulmonary primary: the mass originated in the thyroid lobe rather than the lung, serum calcitonin was markedly elevated (8460 pmol/L), and FNA cytology demonstrated morphological features consistent with MTC, including salt‐and‐pepper chromatin and diffuse TTF‐1 nuclear positivity with strong synaptophysin expression. Taken together, the clinical presentation, imaging characteristics, biochemical profile, and pathological findings established MTC as the diagnosis without the need for further SCLC‐directed investigation. Further imaging with ^68^Ga‐DOTATOC‐PET/CT confirmed a thyroid tumor with metastases to laterocervical lymph nodes (Figure 5A,B) and a distant metastasis in the second thoracic vertebra. Given the locally advanced and metastatic nature of the cancer, it was deemed inoperable after review at an MDT conference. Additional analysis of the thyroid cytology specimen identified a pathogenic variant in exon 16 of the *RET* gene, in addition to a *MYC* gene amplification of unknown significance. However, immunocytochemical staining was negative for ACTH. Somatic analysis of tumor tissue revealed the *RET* gene c.2753T >C, p.Met918Thr mutation. A targeted germline DNA panel, focused on endocrine tumor syndromes including MEN1 and MEN2, showed no pathogenic variants, consistent with a nonhereditary (sporadic) case.

Subsequently, treatment with selpercatinib 160 mg twice daily was initiated. Within 2 days, morning serum cortisol levels before hydrocortisone intake decreased to 180 nmol/L, and ACTH levels normalized. Serum calcitonin and CEA levels dropped to 261 pmol/L and 92 µg/L, respectively. Potassium and glucose levels normalized, allowing potassium supplementation to be discontinued and the dose of spironolactone to be reduced. Metyrapone and hydrocortisone were gradually tapered and eventually discontinued. After 2 weeks, morning serum cortisol was 481 nmol/L, and after 4 weeks midnight salivary cortisol was normal (3.3 nmol/L; ref. <4.7 nmol/L). The rapid biochemical response, with normalization of ACTH after selpinicib initiation and persistent normocortisolemia despite metyrapone discontinuation in combination with the finding of MTC, were regarded as supportive of probable ectopic ACTH production as the underlying mechanism. The patient did not develop any severe side effects except for transient thyroiditis, which possibly developed as a side effect of selpercatinib. A CT scan after approximately 3 months of treatment with selpercatinib revealed partial regression of the tumors, indicating a positive response to treatment; however, the tumor was still deemed unresectable (Figure 5C,D). Hence, it was decided to continue therapy without the addition of metyrapone. At the 10‐month follow‐up, CEA had decreased to 41 μg/L and calcitonin to 275 pmol/L, and a follow‐up CT scan demonstrated continued partial regression of the tumors. The temporal relationship between treatment initiation and biochemical response, including changes in cortisol and calcitonin levels, is illustrated in Figure 3.

## 4. Discussion

MTC is a rare neuroendocrine tumor originating from the parafollicular or C‐cells of the thyroid gland, and the secretion of calcitonin and CEA, constitute crucial biomarkers for its diagnosis and follow‐up [1, 2]. A particularly rare occurrence in MTC is the ectopic production of adrenocorticotropic hormone (ACTH), leading to Cushing’s syndrome. This paraneoplastic syndrome is extremely uncommon, observed in less than 1% of MTC cases, with fewer than 60 cases reported in the literature [6–9].

### 4.1. Clinical Presentation and Pathophysiology

Ectopic ACTH production by MTC results in hypercortisolemia, manifesting as Cushing’s syndrome. Patients may present with classic features such as central obesity, moon face, hypertension, diabetes mellitus, proximal muscle weakness, and hypokalemia [6]. The onset of Cushing’s syndrome can precede, coincide with, or follow the diagnosis of MTC, complicating the clinical picture [3]. In the cases presented, both patients exhibited symptoms consistent with hypercortisolism, including hypertension, hypokalemia, and diabetes mellitus, which prompted further investigation, leading to the diagnosis of MTC with ectopic ACTH production.

Ectopic ACTH production by MTC is a rare but recognized phenomenon. The exact mechanisms are not fully understood, but several hypotheses have been proposed. It is believed that neuroendocrine tumors such as MTC may aberrantly express proopiomelanocortin (POMC), the precursor molecule of ACTH, due to loss of normal transcriptional regulation. This ectopic expression may be triggered by dedifferentiation of tumor cells or epigenetic dysregulation, leading to inappropriate hormone synthesis. Studies have shown that ectopic ACTH‐producing tumors often exhibit expression of POMC mRNA and may lack typical feedback control mechanisms [13].

### 4.2. Diagnostic Challenges

Diagnosing ectopic ACTH syndrome secondary to MTC poses significant challenges. The rarity of this condition necessitates a high index of suspicion, especially in patients presenting with Cushingoid‐related features and a thyroid mass or elevated calcitonin levels [5]. Biochemical confirmation includes elevated serum cortisol and ACTH levels that are not suppressed with high‐dose dexamethasone. Imaging studies, such as CT and positron emission tomography scans, are essential for tumor localization and assessing metastatic spread [2]. In the first case, the patient’s initial presentation with abdominal pain and weight loss led to imaging studies revealing pulmonary nodules and a thyroid mass, ultimately diagnosed as metastatic MTC. The second case involved a patient with progressive weakness and significant weight loss, with imaging revealing an invasive thyroid mass and lymphadenopathy, leading to the diagnosis of MTC. It should also be stressed that rare cases of CRH‐secreting MTCs have been reported. They present with exactly the same symptoms as the ACTH‐secreting MTCs but are ACTH‐negative upon immunohistochemical analysis, thus adding another level of complexity to the clinical workup of MTC patients with overt Cushing’s syndrome [14, 15]. Moreover, ACTH immunoreactivity in ectopic ACTH‐producing tumors, including MTC, is commonly weak and focal rather than diffuse, as repeatedly demonstrated in the literature [10–12].

The diagnostic confirmation of suspected ectopic ACTH production can be particularly challenging in patients presenting with severe hypercortisolism and rapid clinical deterioration. In both cases presented, the extent of the endocrine work‐up was limited by the patients’ critical condition, which precluded further procedures such as CRH stimulation testing, bilateral inferior petrosal sinus sampling, or POMC mRNA analysis. Although these investigations would have provided additional diagnostic certainty, their omission reflects a pragmatic and clinically justified approach in the acute setting. The diagnosis in both cases, therefore, relies on the combined clinical, biochemical, radiological, histopathological, and therapeutic findings, which together form a coherent picture consistent with previously reported cases of ectopic ACTH‐producing MTC. We wish to emphasize that the incomplete workup was a direct consequence of clinical urgency rather than an oversight and that it does not detract from our overall diagnostic confidence in both cases.

### 4.3. Management Strategies

The management of MTC with ectopic ACTH production involves addressing both the malignancy and the resultant hypercortisolism. Surgical resection of the primary tumor and metastatic lesions is the mainstay of treatment; however, in cases where surgery is not feasible due to extensive disease, medical management becomes crucial [3, 5].

**Figure 6:**
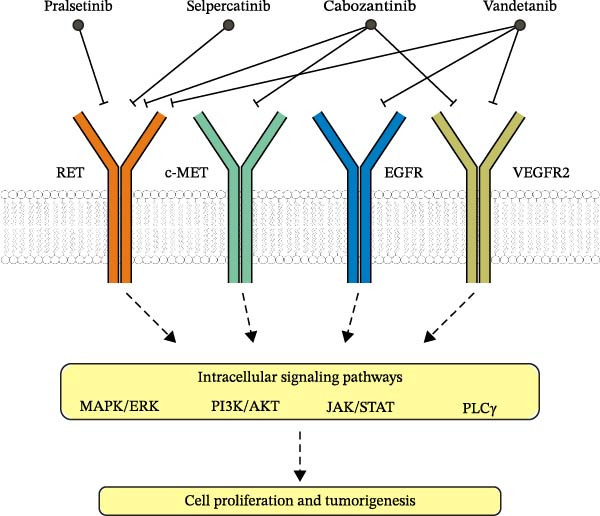
Mechanism of action of TKIs, illustrating the specific target receptors (RET, c‐MET, EGFR, and VEGFR2) of pralsetinib, selpercatinib, cabozantinib, and vandetanib. The diagram shows potential downstream effects on intracellular signaling pathways (MAPK/ERK, PI3K/AKT, JAK/STAT, and PLCγ) leading to the inhibition of cell proliferation and tumorigenesis.

Figure 6 illustrates the potential mechanisms of action of TKIs by targeting specific receptors, including RET, c‐MET, EGFR, and VEGFR2, and their downstream signaling pathways (MAPK/ERK, PI3K/AKT, JAK/STAT, and PLCγ). It is important to note that each TKI has distinct targets and varying downstream effects; for instance, selpercatinib primarily activates MAPK/ERK and PI3K/AKT via RET, while cabozantinib and vandetanib affect a broader receptor range, potentially influencing PLCγ and JAK/STAT signaling. The efficacy of TKIs is further modulated by receptor expression levels and the tumor microenvironment, contributing to variability in therapeutic outcomes. Therefore, the pathways depicted in Figure 6 should be interpreted as potential rather than definitive effects [4, 10].

An important clinical consideration arising from both cases is the risk of acute adrenal insufficiency following initiation of *RET*‐targeted therapy. As demonstrated in Case 1, cortisol levels dropped precipitously to 54 nmol/L within days of starting selpercatinib, necessitating urgent hydrocortisone replacement. Clinicians should anticipate this response and proactively plan for cortisol monitoring and replacement therapy at the time of targeted therapy initiation. Close collaboration between the medical oncologist and endocrinologist is therefore essential in the management of these patients.

The TKIs targeting the *RET* proto‐oncogene, such as vandetanib and cabozantinib, have shown efficacy in controlling tumor progression and associated hormonal syndromes [3]. In both cases, using the selective RET inhibitor selpercatinib rapidly decreased cortisol levels and improved clinical symptoms, highlighting its effectiveness in managing ectopic ACTH production in MTC [16]. Medical management of hypercortisolism includes the use of steroidogenesis inhibitors, like metyrapone, to control cortisol levels. In the first case, metyrapone was initiated to manage hypercortisolism, with the subsequent addition of selpercatinib leading to the normalization of cortisol levels [1, 6]. In the second case, metyrapone was also used initially, with selpercatinib introduced later, resulting in rapid improvement of hypercortisolism [1, 6]. The complicated pathology of ACTH producing MTC as a rare disease and due to the lack of clinical studies about this topic underscore the importance of a multidisciplinary approach involving endocrinologists, oncologists, pathologists, and surgeons in the management of these patients [5]. In cases where the patient’s clinical condition is deteriorating and molecular testing results are pending, initiation of a multikinase inhibitor such as vandetanib as bridge therapy is a clinically reasonable option; transition to a selective RET inhibitor can subsequently be made once the mutational result is confirmed.

An important clinical learning point illustrated by both cases is that MTC should always be included in the differential diagnosis of any patient presenting with a neuroendocrine cancer of unknown primary (CUP) accompanied by ectopic Cushing’s syndrome. Ectopic ACTH production is recognized in a range of neuroendocrine malignancies, including SCLC, Merkel cell carcinoma, and pancreatic neuroendocrine tumors. MTC and Merkel cell carcinoma may occasionally present as neuroendocrine CUP, and failure to consider MTC in this context can significantly delay access to appropriate targeted therapy. In any such patient, serum calcitonin measurement and thyroid ultrasound should be performed as part of the initial workup—even when no obvious thyroid lesion is apparent clinically, as illustrated by Case 1, where a small thyroid primary was identified only on dedicated ultrasonography.

Regarding genetic counseling, germline RET testing should be performed in all newly diagnosed MTC cases irrespective of clinical presentation, as both sporadic and hereditary forms may appear phenotypically similar at diagnosis. Although both cases presented here were confirmed to be sporadic, identification of a hereditary form (MEN2A or MEN2B) should prompt cascade genetic testing of first‐degree family members, offering the opportunity for early detection and potentially curative intervention prior to the development of advanced disease.

It remains unclear whether the observed suppression of ACTH and cortisol levels was primarily due to direct pharmacological effects of RET inhibition on hormone production or secondary to tumor shrinkage. It is possible that both mechanisms contributed to the hormonal response, as suggested in recent case reports and preclinical studies of RET‐driven neuroendocrine tumors [16, 17].

### 4.4. Prognostic Implications

The presence of ectopic ACTH production in MTC is associated with aggressive clinical traits and poor prognosis [18]. Hypercortisolism contributes to increased morbidity and can complicate the management of the underlying malignancy [2, 3]. Early recognition and prompt treatment of both the tumor and the hormonal syndrome are essential to improve outcomes. The advent of targeted therapies, such as selective RET inhibitors, offers new avenues for treatment, potentially improving survival and quality of life for these patients [3, 19]. Managing hypercortisolism is the most critical factor in reducing mortality, with previous literature suggesting that bilateral adrenalectomy is often necessary [10, 15]. Bilateral adrenalectomy represents an important therapeutic option in cases of refractory or life‐threatening hypercortisolism, and its potential benefit should not be underestimated—uncontrolled hypercortisolism carries a greater immediate mortality risk than progressive but indolent MTC. In Case 1, the rapid and sustained biochemical response following initiation of selpercatinib, with cortisol levels remaining well‐controlled at 6‐month follow‐up, meant that bilateral adrenalectomy was not deemed necessary. In Case 2, given the locally advanced and inoperable nature of the tumor, the equally rapid normalization of cortisol following selpercatinib initiation, sustained at 10‐month follow‐up without metyrapone, similarly rendered adrenalectomy unnecessary at this stage. However, bilateral adrenalectomy should be regarded as an important rescue option in patients who do not achieve adequate cortisol control with medical therapy or targeted treatment.

Due to the rarity of ACTH‐producing MTC, correlations between tumor grade and *RET* genotypes remain unknown, and the genetic basis for ectopic ACTH production is not yet understood. Previous studies have identified reduced CpG methylation in the promoter region of POMC, the precursor to ACTH, in ACTH‐secreting pancreatic neuroendocrine tumors [20]. Further genetic studies are needed to determine whether this epigenetic alteration is also present in ACTH‐producing MTCs.

## 5. Conclusion

Ectopic ACTH production by MTC, leading to Cushing’s syndrome, is a rare but significant clinical entity that requires a high index of suspicion for diagnosis. Comprehensive biochemical evaluation and imaging studies are essential for accurate diagnosis and staging. Management involves addressing both the malignancy and the resultant hypercortisolism, with surgical resection being the primary treatment modality when feasible. MTC has emerged as a model disease for precision oncology among rare endocrine tumors. The majority of cases harbor somatic or germline RET mutations, and the availability of potent, selective RET inhibitors— selpercatinib and pralsetinib—has fundamentally transformed the therapeutic landscape. In the specific context of ectopic ACTH‐producing MTC, prompt molecular testing and early access to RET‐targeted therapy are now the critical determinants of clinical outcome, both for tumor control and for resolution of life‐threatening hypercortisolism. A multidisciplinary approach is crucial to optimize patient outcomes in this complex clinical scenario.

## Funding

No funding was received for this manuscript.

## Ethics Statement

Written informed consent was obtained from both patients for genetic testing, publication of clinical data, and use of anonymized images.

## Conflicts of Interest

The authors declare no conflicts of interest.

## Data Availability

The data that support the findings of this study are available upon request from the corresponding author. The data are not publicly available due to privacy or ethical restrictions.
